# Assessment of life factors affecting the experience of depressive symptoms in adolescents: a secondary analysis using the Korea Youth Risk Behavior Survey

**DOI:** 10.1186/s13034-021-00407-0

**Published:** 2021-09-24

**Authors:** Jongha Lee, Changsu Han, Young-Hoon Ko, Moon-Soo Lee, Ho-Kyoung Yoon

**Affiliations:** 1 Department of Psychiatry, Korea University Ansan Hospital, 123, Jeokguem-ro, Danwon-gu, Ansan, Gyeonggi-do 15355 Republic of Korea; 2 Department of Psychiatry, Korea University Guro Hospital, 148, Gurodong-ro, Guro-gu, Seoul, 08308 Republic of Korea

**Keywords:** Adolescents, Depressive mood, Health risk behaviors, Physical activity, Internet use time, KYRBS

## Abstract

**Background:**

Adolescents may experience several changes in their lifestyle, such as social activity and school life, which makes them vulnerable to developing a depressive disorder. Therefore, the present study aimed to identify the factors affecting the experience of depressive symptoms during adolescence.

**Methods:**

We conducted a secondary analysis using the 2019 Korean Youth Risk Behavior Web-based survey data, including a total of 57,303 middle and high school students selected from among 400 schools. Factors such as dietary habits, physical activity levels, time spent studying, duration of internet use, and other health risk behaviors were included in the analysis. Logistic regression analysis was performed to identify factors that predict the risk of experiencing depressive symptoms.

**Results:**

The perceived stress of Korean adolescents showed a tendency to increase with age, and high school seniors and girls were more likely to report depressive symptoms. Perceived health status, academic performance, time spent studying, physical activity, duration of internet use, and effort to control weight were associated with individual experiences of depressive symptoms.

**Conclusion:**

We identified factors that influence the experience of depressive symptoms in adolescents. Our results suggest the possibility that the purpose of students’ physical activities and their leisure activity preferences may be related to their emotional status. We suggest that activities that are appropriate to the culture and lifestyle of adolescents should be recommended to reduce the occurrence of depressive symptoms.

**Supplementary Information:**

The online version contains supplementary material available at 10.1186/s13034-021-00407-0.

## Background

Adolescence is an important period of rapid physical development that requires the individuals to adapt to a new environment [1]. Adolescents may experience changes in school activities, interpersonal relationships, after-school activities, eating habits, and physical activity levels. These changes are associated with a high risk of the onset of mental disorders such as depressive disorder, anxiety disorder, and substance use disorder [2–4]; in particular, the prevalence of major depressive disorder among adolescents appears to be increasing, with one US-based study showing an increase from 8.69 in 2005 to 12.66% in 2015 [5]. The onset of major depressive episodes during adolescence can not only affect adolescents’ daily life but also one’s quality of life in adulthood; thus, assessment of risk factors, early detection, and intervention become important [6].

Several studies have been conducted in order to identify the various factors that influence the prevalence of depressive disorders among adolescents [2, 3, 7–12]. Eating habits are one such factor associated with mental health problems in adolescence. Adolescents with irregular eating habits, such as skipping meals, were more likely to report depression symptoms and high perceived anxiety [2, 4, 8]. Additionally, food insecurity is associated with poor mental health, which in turn leads to lower academic achievement [13]. However, the number of teenagers who skip breakfast in many countries continues to increase, and has been recognized as a social problem. A study conducted in Korea suggested that the most common reason teenagers skip breakfast is the lack of time resultant from getting ready for school or private tutoring sessions [14]. Physical activity is another factor that positively affects mental health among adolescents. Physical activity guidelines for Americans recommend that adolescents aged 6–17 years engage in at least 60 min of moderate-to-vigorous exercise per day [15].

Internet use in adolescents is also known to affect mental health [9, 10]. Unlike in the past, adolescents and young adults now rely on the internet in many aspects of their daily life, including school activities, interpersonal relationships, information acquisition, and leisure activities. Despite the convenience of the internet, the excessive use of social networking services (SNS) and online game addiction among teenagers have been evaluated as negative factors affecting their mental health. Although it is still difficult to define the optimal time duration for internet use among adolescents, it is generally advised that this is limited to 2 h a day [16, 17]. However, some argue that this recommendation is virtually impossible [18], while another study reported that even 4 h of internet use per day does not negatively affect mental health [19]. Furthermore, family environments can also affect the mental health of adolescents. Adolescents from multicultural families or immigrant families, in particular, may be more likely to experience depressive and anxiety symptoms and are at a high risk of developing mental illness [11, 12, 20]. In addition, academic performance, socioeconomic status, gender, smoking, alcohol use, and other characteristics of daily life also impact adolescents’ mental health and have been correlated with depressive disorder, anxiety disorder, and substance use disorder [7, 13, 21].

As highlighted above, previous studies have focused on individual factors that affect the mental health of adolescents; however, comprehensive studies focusing on multiple variables, including health risk behavior, family structure, physical activity, and internet usage, are lacking. In the present study, we aimed to identify the factors associated with the onset of a major depressive disorder in adolescence and to make recommendations for a healthy lifestyle for adolescents.

## Methods

### Participants

This study analyzed secondary data obtained from the 15th (2019) Korean Youth Risk Behavior Web-based Survey (KYRBS). The KYRBS is conducted annually by the Korean Disease Control and Prevention Agency (KDCA) to evaluate the physical and mental health status of Korean middle and high school students [22]. The KYRBS adopts a multi-stage cluster sampling design to obtain a representative sample of Korean adolescents, by dividing 17 provinces nationwide into 44 regions according to the size of the city and then selecting middle school (grades 7–9) and high school (grades 10–12) students from 800 schools. Both private and public schools were included in the survey; for middle schools, the sample number of schools was allocated according to gender (male, female, co-education); for high schools, the sample number of schools was allocated according to gender (male, female, co-education) and the purpose of school education (academic high school and specialized vocational high school).

The KYRBS is an anonymous self-response online survey, which is completed by the selected students while sitting in their school’s computer room under the supervision of trained teachers. Participating students and their parents received informed written consent from the individual school. After receiving a detailed explanation regarding the purpose of the survey and the procedure from a trained teacher, students were asked to decide on whether they wanted to participate. Parents were asked to provide passive consent on behalf of their children and were instructed to verbally notify the teachers if they did not want their children to participate. Written informed consent was obtained from all the participants. Detailed information regarding the research design and methods of the KYRBS can be found in a previous paper [22]. In the 2019 KYRBS, 60,100 people from 800 schools (400 middle schools and 400 high schools) were selected as participants, of which 57,303 (95.3%) agreed to participate in the survey. Since the KYRBS was conducted as an online survey, there were no non-response items in the original data; however, logical errors and outliers were treated as missing values. For the purpose of this study, the data file for the SPSS program provided by KDCA was used for analyses.

### Variables

#### Demographic information

We analyzed demographic data, including gender, age, size of the city, school type, grade, educational background, perceived academic performance, perceived household economic status, type of residence, and parents’ nationality. The size of the city was categorized as big, small to medium, or small, based on geographical accessibility, number of schools, population size, and living environment. Perceived academic performance and perceived household economic status were classified into five categories: upper, upper-middle, middle, lower-middle, and lower. The type of residence was categorized as: (1) living in a parent’s house, (2) living in a relative’s house, (3) lodging or living alone, (4) living in a dormitory, and (5) living in a childcare facility.. Information on the family, including parental nationality, was available only if the participants agreed to answer the question. In this study, we classified nationalities as Korean or other nationalities.

#### Health risk behavior and health status

We analyzed the number of days the participants ate breakfast during the week, the number of days they engaged in physical activity for more than 60 min a day during the week, and the amount of time spent sitting on weekdays/weekend to study and use the internet. Concerning physical activity, we assessed the number of days on which their heart rate was higher than usual or they engaged in 60 min or more of physical activity, which left them breathless, regardless of the type of physical activity. The participants were asked to report separately the average time spent sitting for studying on weekdays/weekends, including during school time. Considering that the participants spent approximately 6 h at school (class time: middle school students, about 300 min a day; high school students, about 350 min a day), time spent sitting on weekdays was classified as follows: < 240 min, 240–359 min, 360–479 min, 480–599 min, 600–719 min, and ≥ 720 min; over the weekend, time spent sitting for studying was categorized as none, 1–119 min, 120–239 min, 240–359 min, 360–479 min, and ≥ 480 min. Duration of internet use was classified as none, 1–60 min, 61–120 min, 121–180 min, and ≥ 180 min. The subjective perception of their own health status was answered with five responses: “very healthy,” “healthy,” “normal,” “unhealthy,” and “very unhealthy.” In addition, the subjective perception of body shape was evaluated as follows: “very skinny,” “skinny,” “average,” “obese,” and “very obese.” Health risk behaviors such as smoking, alcohol use, habitual drug/substance use, and sexual activity were also evaluated. Participants also answered whether they had received hospital-based treatment for any violent incidents within 1 year; this was categorized as either “yes” or “no” for the regression analysis.

#### Mental health status

The perceived stress level and experience of depressive symptoms were analyzed in this study. The former was classified into five responses: “very much,” “a lot,” “a little,” “not much,” and “not at all,” and the latter was evaluated using the question: “Have you experienced a feeling of sadness or despair that has interrupted your daily life for at least 2 weeks in the last 12 months?” Unfortunately, the severity of depressive symptoms was not recorded in the original data. Although there are limitations to clinically diagnosing a participant as suffering from a depressive disorder based on these questions, the presence of depressive mood and deterioration of functioning on most days during a period of 2 weeks can suggest the possible occurrence of depressive episodes.

### Statistical analyses

In this study, students who provided insufficient answers were excluded; finally, data from 46,206 participants were included in the analysis. All statistical analyses were performed using SPSS software (version 25.0; IBM Corp., Armonk, NY, 2019), and *p*-values of less than 0.05 were considered statistically significant. The variables (demographic information, health risk behaviors, sitting time, and physical/mental health status) were compared by grade groups using a one-way analysis of variance (ANOVA) and a chi-square test. Logistic regression analyses that included all variables were performed to confirm the relationship between individual variables and experiences of depressive symptoms. Binary regression analysis was performed using the experience of depressive symptoms as the dependent variable, followed by a regression analysis of the statistically significant variables and the experience of depressive symptoms. The results of the logistic regression were reported as unadjusted and adjusted odds ratios with 95% confidence intervals.

### Ethics statement

The KYRBS was conducted by the Ministry of Education, Ministry of Health and Welfare, and the KDCA, and contained nationally approved statistical data [22]. The raw data used in this study were approved for use through the KDCA website. These data were provided along with a unique number that cannot be identified without the participant’s personal information, thereby ensuring the participant’s anonymity. The protocol of this secondary analysis study was approved by the Institutional Review Board of the Korea University Medical Center, Ansan Hospital, Gyeonggi-do, Korea (No. 2020AS0309).

## Results

### Sociodemographic characteristics, health-risk behaviors, and physical/mental health status

The major findings are presented in Table 1, and information on all variables included in this study is presented in Additional file 1: Table S1. In each grade group, more boys participated than girls; however, this difference was not statistically significant. The proportion of co-educational schools was the highest among all middle and high schools, although, their proportion was lower in the case of high schools than in middle schools. Less than 3% of the adolescents in the sample belonged to multicultural families, and most of them were in grade 7. Compared to high school students, middle school students perceived their academic achievement and family economic status to be higher. In contrast, as the grade level increased, the number of students who perceived their health status and body shape increased negatively. The percentage of high school students who perceived themselves as being (very) unhealthy or (very) obese was higher than that of middle school students. More than 50% of the participants had tried to control their weight, and these efforts were primarily aimed at weight loss, followed by weight maintenance and weight gain. As they grew older, the percentage of adolescents who were physically active for more than 60 min a day tended to decrease. Additionally, the duration of sitting for studying increased with grade level, as was clear among the high school student participants (grade 9: 425.80 ± 237.63, grade 10: 520.05 ± 263.81). Perceived stress (“a lot” or “very much”) also tended to increase with age, and the number of students who experienced depressive symptoms within the past year was the highest during the third year of high school (Table 1). Further, the number of students who smoked and consumed alcohol increased with age, with 58.7% of students in the third year of high school reporting that they had engaged in drinking. Of those in the first year of middle school, 1.2% had engaged in sexual activity and this number gradually increased with increasing grade level; in the third year of high school, 9.9% of the students reported having engaged in sexual activity (Additional file 1: Table S1).

**Table 1:** Sociodemographic characteristics

Variables	Grade 7	Grade 8	Grade 9	Grade 10	Grade 11	Grade 12	*p*-value
	(*n*: 7975)	(*n*: 7723)	(*n*: 8014)	(*n*: 7498)	(*n*: 7242)	(*n*: 7754)	
Age (in years)	12.50 ± 0.51	13.52 ± 0.52	14.51 ± 0.52	15.51 ± 0.52	16.50 ± 0.52	17.47 ± 0.52	
Gender, Girls	3879 (48.6)	3803 (49.2)	3865 (48.2)	3619 (48.3)	3544 (48.9)	3723 (48.0)	0.637
School type							< 0.01
Co-educational school	5878 (73.7)	5664 (73.3)	5900 (73.6)	4377 (58.4)	4204 (48.1)	4491 (57.9)	
Boys school	1079 (13.5)	1074 (13.9)	1095 (13.7)	1532 (20.4)	1498 (20.7)	1604 (20.7)	
Girls school	1018 (12.8)	985 (12.8)	1019 (12.7)	1589 (21.2)	154 (21.3)	1659 (21.4)	
Multicultural family							< 0.01
Yes	170 (2.8)	130 (2.4)	103 (1.9)	62 (1.3)	58 (1.3)	57 (1.3)	
No	5803 (97.2)	5213 (97.6)	5213 (98.1)	4617 (98.7)	4256 (98.7)	4371 (98.7)	
Perceived academic performance							< 0.01
Upper	1468 (18.4)	1183 (15.3)	1323 (16.5)	737 (9.8)	702 (9.7)	829 (10.7)	
Upper middle	2493 (31.3)	2068 (26.8)	2083 (26.0)	1753 (23.4)	1665 (23.0)	1857 (23.9)	
Middle	2509 (31.5)	2238 (29.0)	2093 (26.1)	2343 (31.2)	2379 (32.9)	2521 (32.5)	
Lower middle	1143 (14.3)	1616 (20.9)	1768 (22.1)	1861 (24.8)	1773 (24.5)	1844 (23.8)	
Lower	362 (4.5)	618 (8.0)	747 (9.3)	804 (10.7)	723 (10.0)	703 (9.1)	
Perceived household economic status							< 0.01
Upper	1303 (16.3)	965 (12.5)	874 (10.9)	652 (8.7)	597 (8.2)	578 (7.5)	
Upper middle	2635 (33.0)	2350 (30.4)	2344 (29.2)	2071 (27.6)	1894 (26.2)	1946 (25.1)	
Middle	3425 (42.9)	3638 (47.1)	392 (48.8)	3761 (50.2)	3615 (49.9)	3952 (51.0)	
Lower middle	542 (6.8)	661 (8.6)	751 (9.4)	842 (11.2)	933 (12.9)	1055 (13.6)	
Lower	70 (0.9)	109 (1.4)	133 (1.7)	172 (2.3)	203 (2.8)	223 (2.9)	
Dietary behavior^a^ (days)	4.28 (± 2.74)	4.04 (± 2.75)	3.95 (± 2.77)	4.02 (± 2.68)	3.83 (± 2.68)	4.01 (± 2.70)	< 0.01
Perceived health status							< 0.01
Very healthy	2438 (30.6)	2134 (27.6)	2292 (28.6)	1932 (25.8)	1714 (23.7)	1860 (24.0)	
Healthy	3736 (46.8)	3544 (45.9)	3527 (44.0)	3401 (45.4)	3151 (43.5)	3200 (41.3)	
Normal	1528 (19.2)	1667 (21.6)	1715 (21.4)	1604 (21.4)	1753 (24.2)	1933 (24.9)	
Unhealthy	263 (3.3)	358 (4.6)	455 (5.7)	532 (7.1)	575 (7.9)	712 (9.2)	
Very unhealthy	10 (0.1)	20 (0.3)	25 (0.3)	29 (0.4)	49 (0.7)	49 (0.6)	
Perception of body shape							< 0.01
Very skinny	332 (4.2)	336 (4.4)	359 (4.5)	341 (4.5)	293 (4.0)	285 (3.7)	
Skinny	1802 (22.6)	1756 (22.7)	1770 (22.1)	1582 (21.1)	1465 (20.2)	1480 (19.1)	
Average	2944 (37.5)	2898 (37.5)	2965 (37.0)	2680 (35.7)	2572 (35.5)	2727 (35.2)	
Obese	2395 (30.0)	2309 (29.9)	2465 (30.8)	2399 (32.0)	2682 (34.6)	2682 (34.6)	
Very obese	452 (5.7)	424 (5.5)	455 (5.7)	496 (6.6)	514 (7.1)	580 (7.5)	
Physical activity^b^ (in days)	2.32 ± 2.21	2.26 ± 2.16	2.20 ± 2.19	1.83 ± 2.02	1.84 ± 2.01	1.69 ± 2.00	< 0.01
Time spent studying (WD^c^) (in mins)	376.68 ± 242.87	415.80 ± 242.87	425.80 ± 237.63	520.05 ± 263.81	516.83 ± 251.16	550.70 ± 252.37	< 0.01
Time spent studying (WK^d^) (in mins)	148.56 ± 155.73	186.37 ± 178.95	191.13 ± 187.34	266.74 ± 225.93	276.76 ± 232.37	339.08 ± 266.73	< 0.01
Duration of Internet use (WD^c^) (in mins)	111.75 ± 112.57	122.82 ± 122.45	117.96 ± 120.20	104.29 ± 109.48	103.07 ± 110.23	97.73 ± 105.12	< 0.01
Duration of Internet use (WK^d^) (in mins)	186.55 ± 163.00	208.39 ± 174.33	202.43 ± 176.93	189.43 ± 161.08	174.93 ± 161.61	163.74 ± 152.89	< 0.01
Perceived level of stress							< 0.01
Very much	643 (8.1)	754 (9.8)	820 (10.2)	822 (11.0)	874 (12.1)	976 (12.6)	
A lot	2021 (25.3)	2156 (27.9)	2143 (26.7)	2166 (28.9)	2175 (30.0)	2264 (29.2)	
A little	3276 (41.1)	3204 (41.5)	3407 (42.5)	3203 (42.7)	2991 (41.3)	3223 (41.6)	
Not much	1625 (20.4)	1307 (16.9)	1353 (16.9)	1044 (13.9)	989 (13.7)	1057 (13.6)	
Not at all	410 (5.1)	302 (3.9)	291 (3.6)	263 (3.5)	213 (2.9)	234 (3.0)	
Depressive symptoms, (yes)	1752 (22.0)	2075 (26.9)	2228 (27.8)	1993 (26.6)	2024 (27.9)	2294 (29.6)	< 0.01

Each number is presented as the mean (standard deviation) or sample size (ratio by group)

^a^Dietary behavior: Number of days per week on which participants ate breakfast

^b^Physical activity: number of days per week participants engaged in physical activity for at least 60 min per day

^c^WD: weekday

^d^WK: weekend

### Variables predicting the experience of depressive symptoms

We identified the individual variables that influence the experience of depressive symptoms using univariable logistic regression analyses. The risk of developing depressive symptoms was found to be the highest among students from girls’ schools, followed by co-educational schools and boys’ schools; girls were at a higher risk of experiencing depressive symptoms than boys. Furthermore, the higher the grade level, the higher the risk of developing depressive symptoms. Those with low academic achievement also faced a higher risk of depression than those with high academic achievement. Belonging to a multicultural family was not a significant factor influencing the experience of depression; however, adolescents who did not live with their parents were at a higher risk of depression than those living with their parents. Negative perceptions of physical health status, body shape, and efforts to control weight, were also found to increase the risk of developing depressive symptoms. Students who ate breakfast more than 5 days a week had a lower risk of developing depressive symptoms; however, those who ate breakfast on only 1 day during the week were at a higher risk than those who never ate breakfast. While engaging in physical activity once a week was associated with an increased risk of experiencing depressive symptoms, engaging in physical activity for more than 2 days a week was not found to be statistically significant. The risk was also higher in the group that spent more than 12 h studying on weekdays [odds ratio (OR) = 1.178], whereas those who spent less than 4 h studying on weekdays had a lower risk of experiencing depressive symptoms (1–119 min: OR = 0.876 and 120–239 min: OR = 0.928). Using the internet for up to 1 h on weekdays and 3 h on weekends was associated with a low risk of developing depressive symptoms. Alternately, if the internet usage was over 3 h on weekdays, the risk increased. Alcohol drinking, smoking, drug/substance use, sexual activity, and history of hospital-based treatment for violent events in the past year were all identified as factors that increased the risk of experiencing depressive symptoms.

We also performed a multivariable logistic regression analysis with all the aforementioned statistically significant variables, except for multicultural family and city type. After controlling for each variable, perceived physical health status, perceived academic performance, time spent studying or for internet use, physical activity, and other health risk behaviors showed significant results. Increasing physical activity levels [adjusted odds ratio (aOR) = 1.212–1.625)] and, more than 2 h on the weekends, were both identified as being associated with the risk of experiencing depressive symptoms. In contrast, using the internet on weekends was found to be associated with a lower risk of experiencing depressive symptoms. The main findings of the regression analysis including physical activity, study time, and internet use are presented in Table 2, and all findings are presented in Additional file 1: Table S2.

**Table 2:** Logistic regression analyses of factors associated with the experience of depressive symptoms

Variables	Unadjusted model				Adjusted model^a^			
	OR	95% Cl for OR		p-value	OR	95% Cl for OR		p-value
Gender								
Boys	1							
Girls	1.879	1.802	1.959	< 0.001	1.998	1.881	2.122	< 0.001
School grade								
Middle 1st grade	1				1			
Middle 2nd grade	1.305	1.213	1.404	< 0.001	1.123	1.038	1.215	< 0.01
Middle 3rd grade	1.368	1.273	1.470	< 0.001	1.098	1.015	1.188	< 0.05
High 1st grade	1.286	1.195	1.384	< 0.001	0.924	0.849	1.006	0.067
High 2nd grade	1.378	1.280	1.483	< 0.001	0.867	0.795	0.945	< 0.01
High 3rd grade	1.492	1.389	1.604	< 0.001	0.886	0.812	0.966	< 0.01
Perceived health status								
Very healthy	1				1			
Healthy	1.429	1.352	1.511	< 0.001	1.377	1.297	1.463	< 0.001
Normal	2.326	2.189	2.473	< 0.001	2.115	1.975	2.264	< 0.001
Unhealthy	3.897	3.577	4.247	< 0.001	3.406	3.095	3.748	< 0.001
Very unhealthy	6.393	4.740	8.623	< 0.001	6.198	4.474	8.585	< 0.001
Dietary behavior^b^								
None	1				1			
1 day	1.145	1.049	1.248	< 0.01	1.086	0.989	1.193	0.086
2 days	1.020	0.939	1.107	0.645	0.992	0.908	1.085	0.866
3 days	1.007	0.925	1.095	0.880	0.970	0.886	1.063	0.514
4 days	0.983	0.898	1.076	0.711	0.975	0.884	1.075	0.608
5 days	0.904	0.837	0.977	< 0.05	0.948	0.872	1.031	0.213
6 days	0.820	0.751	0.894	< 0.001	0.895	0.815	0.983	< 0.05
7 days	0.671	0.632	0.712	< 0.001	0.793	0.742	0.847	< 0.001
Physical activity^c^								
None	1				1			
1 day	1.136	1.067	1.209	< 0.001	1.212	1.133	1.297	< 0.001
2 days	1.007	0.945	1.073	0.836	1.181	1.102	1.265	< 0.001
3 days	1.014	0.948	1.085	0.682	1.297	1.203	1.398	< 0.001
4 days	0.943	0.864	1.030	0.193	1.292	1.173	1.425	< 0.001
5 days	0.951	0.869	1.040	0.271	1.328	1.201	1.467	< 0.001
6 days	0.985	0.856	1.134	0.837	1.446	1.241	1.685	< 0.001
7 days	1.054	0.965	1.151	0.244	1.625	1.469	1.797	< 0.001
Time spent studying (WD^d^)								
360–479 min	1				1			
< 240 min	0.990	0.920	1.065	0.777	1.149	1.062	1.243	< 0.01
240–359 min	1.079	0.986	1.182	0.100	1.194	1.083	1.316	< 0.001
480–599 min	1.022	0.949	1.101	0.565	1.015	0.937	1.099	0.715
600–719 min	1.077	0.999	1.161	0.052	1.062	0.978	1.154	0.153
> 720 min	1.178	1.095	1.268	< 0.001	1.125	1.033	1.226	< 0.01
Time spent studying (WK^e^)								
None	1				1			
1–119 min	0.876	0.812	0.944	< 0.001	1.014	0.934	1.100	0.743
120–239 min	0.928	0.864	0.996	< 0.05	1.102	1.018	1.193	< 0.05
240–359 min	0.991	0.919	1.070	0.824	1.217	1.116	1.327	< 0.001
360–479 min	0.985	0.901	1.077	0.744	1.267	1.144	1.404	< 0.001
> 480 min	1.121	1.039	1.210	< 0.01	1.518	1.380	1.670	< 0.001
Duration of internet use (WD^d^)								
None	1				1			
1–60 min	0.885	0.834	0.940	< 0.001	1.025	0.949	1.107	0.531
61–120 min	0.954	0.897	1.014	0.134	1.057	0.974	1.147	0.184
121–180 min	1.057	0.987	1.132	0.115	1.042	0.950	1.144	0.381
> 180 min	1.344	1.259	1.434	< 0.001	1.174	1.068	1.292	< 0.05
Duration of internet use (WK^e^)								
None	1				1			
1–60 min	0.799	0.739	0.865	< 0.001	0.866	0.788	0.952	< 0.01
61–120 min	0.818	0.761	0.879	< 0.001	0.895	0.819	0.979	< 0.05
121–180 min	0.817	0.762	0.877	< 0.001	0.894	0.817	0.979	< 0.05
> 180 min	1.000	0.947	1.057	0.993	0.929	0.854	1.010	< 0.01

*CI* confidence interval, *OR* odds ratio

^a^Adjusted model for all variables except multicultural family and city type

^b^Dietary behavior: Number of days per week on which participants ate breakfast

^c^Physical activity: Number of days per week participants engaged in physical activity for at least 60 min per day

^d^WD: weekday

^e^WK: weekend

## Discussion

In this study, we identified factors related to the experience of depressive symptoms among adolescents. Consistent with the findings of previous studies [3, 20, 23–27], gender, age, academic achievement, family economic status, type of residence, physical activity level, health status, perception of body shape, and study time were found to be related to the presence of depressive symptoms among adolescents. Smoking, alcohol use, being a victim of violence, and teenage sexual experiences, which are considered risk factors for adolescent depressive disorder, were also identified as factors that increased the risk of experiencing depressive symptoms in this study; among these, being a victim of violence was found to be the greatest risk factor (aOR = 1.516–2.149). However, belonging to a multicultural family was not found to be a statistically significant factor influencing the presence of depressive symptoms. Further, the perception of belonging to a middle-income family lowered the risk of developing depressive symptoms.

Physical health is closely related to mental health, and physically ill patients are at a high risk of developing a depressive disorder [24, 28]. Moreover, in adolescence, individuals’ perception of their own body shape affects their self-esteem and mental health [24]. That is, a negative perception of one’s body shape is a risk factor for developing mental disorders such as depressive disorder [29]. In this study, adolescents who thought that they were (very) obese or (very) skinny were at a high risk of experiencing depressive symptoms in the univariable analysis; however, in the multivariable analysis, only the perception that one’s body was (very) skinny was found to be a risk factor. Additionally, efforts aimed at controlling their weight were also evaluated as a risk factor; we judged that such efforts made by adolescents to maintain their socially preferred skinny body would have served as a stress factor for them. We also found that eating breakfast regularly influenced the experience of depressive symptoms: adolescents who had breakfast more than 5 days a week were at a significantly lower risk of experiencing depressive symptoms.

Academic achievement is considered critical for students, and college entrance is a major stress factor, especially among high school students in Korea. In a previous study conducted in Korea, subjective poor academic performance was a risk factor for adolescent suicide and depressive disorder [30]. Korean adolescents want to have a stable job in the future, and they recognize that admission to a good university is paramount for this. Regardless of their preferences or intentions, they spend a lot of time studying, and their self-esteem and emotional satisfaction are affected by their academic performance. Korean students worry about not achieving good academic performance, and when they get lower grades than their expectations, they may perceive themselves as “losers.” Concerning adolescents’ social environment, academic achievement and time spent studying were found to be risk factors for developing depressive symptoms. In the univariable regression analysis, those who were in the third year of high school were found to be at a higher risk of experiencing depressive symptoms; however, in the multivariable regression analysis, the risk tended to decrease as the grade level increased. Instead, students with lower academic achievement were at a higher risk of experiencing depressive symptoms than those with higher academic achievement in the same analysis. Given these findings, we believe that the risk of developing depressive symptoms increases as the grade level increases, due to stress caused by college entrance exams. Similar results have been reported in studies conducted in Finland and Iran [31, 32]. Students with low academic performance showed high levels of anxiety and depression. It can also be interpreted in the same context that time spent studying is related to depressive symptoms. Adolescents who spent less time studying than the average number of school hours on weekdays had a higher risk of developing depressive symptoms. In the multivariable regression analysis, adolescents who studied for less than 4 h or more than 12 h on weekdays were at a greater risk of developing depressive symptoms. The higher risk associated with less sitting time for studying may be related to school refusal. Students with school refusal are at a higher risk of experiencing depressive disorder and anxiety disorder, and adolescents with depressive disorders have also been reported to experience a decline in functioning at school and in academic achievement [33–35]. Excessive study hours can also be a stress factor for adolescents, given that such a routine makes it difficult for them to get enough rest. Adolescents who spent more than 2 h studying per day on weekends were more likely to report more depressive symptoms. Therefore, as indicated by the findings, spending 6–12 h studying, including school hours, on weekdays and less than 2 h on weekends can be appropriate and helpful in reducing the risk of depressive episodes.

In addition to the findings described above, several findings ddiffered from those of previous studies. For instance, existing literature states that regular physical activity is associated with low levels of depression and anxiety [2, 3]. However, this study with Korean adolescents showed the opposite results. In the univariable regression analysis, adolescents who exercised 1 day per week were at a higher risk of experiencing depressive symptoms than those who did not exercise at all (OR = 1.136). In the multivariable logistic regression, after adjusting for other variables, the risk of experiencing depressive symptoms increased with an increase in the number of days of physical activity (aOR = 1.181–1.625). Caution is needed to interpret this result. First, the KYRBS is a cross-sectional survey, and there is a limit to determining causality. The presence of depressive symptoms and the frequency of physical activity affect each other, and the possibility that depressive symptoms may result in decreased physical activity cannot be excluded. In addition, it was thought that the average frequency of physical activity among Korean students was low (mean: 2.03 days a week), and the characteristics of the physically active group may have influenced the results. When the group was divided according to whether the effort was made to control weight, the group with the effort to control weight (the group with ECW (effort to control weight), n = 24,363) had a higher frequency of physical activity than the group without effort to control weight (the group without ECW, n = 21,843), and the average number of times of physical activity was 2.30 days and 1.73 days, respectively. About 62% of students who exercised for more than 4 days reported that they were trying to control their weight. Subjective stress was higher in the group with ECW than in the group without ECW, and the rate of reporting experience of depressive symptoms was higher in the group with ECW (29.8% vs 23.4% in the group without ECW). Our results suggest that the purpose of physical activities of Korean adolescents is highly related to efforts to control weight and the possibility that the group with ECW has a risk of experiencing depressive symptoms.

Internet use during the weekend was found to be associated with a lower risk of experiencing depressive symptoms; we believe this is related to the culture of leisure among Korean youth. Korea has a high internet penetration rate, and teenagers can easily access the internet through their smartphones and PCs. In addition, the number of students returning home late after attending private tutoring sessions after school has been rising; consequently, there is limited time available for students to engage in physical activities, outdoor activities, and meeting friends in person. Considering this situation, it is likely that Korean adolescents prefer engaging in indoor activities, such as playing online games, over physical activities to relieve stress. In another study conducted in South Korea, adolescents reported using the internet for pleasure, stress relief, and socializing with friends [36]. Based on these results, we suggest that the favorable and adverse effects of internet use must be reevaluated according to cultural characteristics. Existing studies have mainly focused on the problem of internet use [9, 10, 37, 38]. Internet game addiction, excessive use of SNS, obesity due to reduced physical activity, and decline in basic physical strength can be problematic, but it is not wise to judge internet use only negatively. The internet is being increasingly used as a means to engage in leisure activities, such as playing online games, watching movies, socializing, and acquiring information. Adequate internet use can reduce tension and improve mood in the same way as physical activity. Our results suggest that excessive use of the internet leads to an increased risk of developing depressive symptoms and, consequently, adolescents should have limited access to it, for example, less than 3 h on weekends under the supervision of a caregiver.

Adolescents from multicultural families are at a high risk of exposure to school violence or bullying due to different physical appearances, cultural differences, social discrimination, and prejudice [39, 40]. They may also experience emotional difficulties in many areas of their lives and negatively affect their ego formation process [41, 42]. The proportion of multicultural youth in Korea has been increasing, along with an increase in social interest in emotional health. In this study, we compared adolescents belonging to multicultural and monocultural (Korean) families; however, there was no statistically significant difference between the two groups in their subjective experience of depressive symptoms. This finding might be influenced by the characteristics of multicultural families in Korea. First, in Korea, multicultural families refer to families comprising non-Korean parents; however, in the National Survey of Multicultural Families 2015, more than 80% of multicultural families consisted of Korean husbands and non-Korean wives [41, 43]. Their children are, therefore, likely to have lived in Korea from the time they were born because of which problems due to cultural differences would have been minimized. Findings from previous studies regarding whether multicultural families are a risk factor for depression in Korea are inconsistent [41, 44]. Second, considering that personal factors, such as resilience and parenting attitudes, influence adolescents' levels of school adjustment [44], we judged that these factors may also be related to their mental health. Finally, multicultural families in Korea tend to live in certain regions; in some regions, the proportion of multicultural families accounted for more than 10% of the local population [45]. Thus, the risk of prejudice and discrimination is also thought to be lower in these regions. There is a need for future studies to focus on emotional difficulties among multicultural adolescents based on the characteristics of their parents' nationality, duration of residence in Korea, region of residence, and language ability.

The strengths of this study include the comprehensive evaluation of factors related to the risk of developing depressive symptoms among adolescents. By presenting findings that significantly differ from those of existing studies, we were able to highlight the need to establish a new standard of healthy lifestyle according to Korean youth culture. That is, depending on the adolescents’ living environment, setting an appropriate time limit for studying and internet use may be more beneficial to mental health. Nevertheless, this study had some limitations. First, concerning physical activity, we did not evaluate the type of physical activity performed, students’ spontaneity, and students’ preference for physical activity. The KYRBS includes students who engage in physical activity during their physical education class regardless of their will and students who worked part-time or wanted to be professional athletes. For them, physical activity may negatively affect their emotional states. As mentioned earlier, the fact that a large number of physically active students were trying to control their weight may have had an influence. To more clearly define the causal relationship, future studies must confirm the effect of spontaneity and the purpose of physical activity on the experience of depressive symptoms. Second, this study was conducted with adolescents residing in South Korea, and caution needs to be exercised when interpreting the results. Given that the risk factors related to Korean youth and students' interests are different, it can be difficult to generalize the recommendations regarding the appropriate time for studying and using the internet to all students. Thus, our recommendation will have to be modified based on an individuals’ characteristics and the culture to which they belong. Third, there is a possibility that individual biases were present in the data because participants were asked to provide their subjective perceptions, instead of using objective measures for variables. However, previous studies have shown that subjective perceptions influence emotional states in adolescents [46, 47]. In a previous study conducted in China, the perceived weight was more related to psychological difficulties than the actual weight, and students who described themselves as overweight reported higher levels of anxiety or depressive symptoms [46]. Similarly, in a study of US adolescents, McLaughlin et al. found that lower subjective social status was associated with higher odds of mood disorders, anxiety disorders, disruptive behavior disorders, and substance disorder, whereas an objective indicator, family income, was not related [47]. In line with this, adolescents’ subjective evaluations of socioeconomic status in the present study influenced their emotional status. Fourth, we were unable to suggest the maximum internet use time that can negatively affect the mental health of youth. Given that a previous study indicated that using the internet for more than 7 h a day is related to depressive symptoms [10], further research is needed in this regard. Fifth, we could not distinguish between social drinking and habitual drinking because the KYRBS data have limitations in what concerns drinking habits and alcohol consumption. In the survey, drinking experiences, except experiences of drinking during religious ceremonies and ancestral rites, were collected. The results of this study suggest the effect of drinking experience on the experience of depressive symptoms. Finally, although students reported the subjective experience of depressive symptoms, the severity of these symptoms was not evaluated. There is a limitation in diagnosing adolescents, who report experiencing depressive symptoms, as suffering from a depressive disorder; however, understanding whether they experienced depressive symptoms, along with a decline in functioning, for more than 2 weeks, can be meaningful for assessing their mental health status.

## Conclusion

We identified the factors associated with the experience of depressive symptoms among Korean adolescents. Using the internet for more than 3 h on weekdays and studying for an inappropriate amount of time (less than 6 h on weekdays and more than 2 h on weekends) were found to be associated with a higher risk of experiencing depressive symptoms. Alternately, adequate internet use during the weekends and having breakfast regularly were both found to reduce the risk of experiencing depressive symptoms. Unlike previous studies conducted in other countries, physical activity was identified as a risk factor among Korean adolescents. We argue that this finding reflects Korean adolescents’ ways of managing stress, as well as their culture and lifestyles. The identified risk factors, such as academic achievement and family economic status, are highly related to social awareness, and efforts to reduce these prejudices are needed. Furthermore, to prevent the onset of depressive episodes in adolescents, it is necessary to understand their cultures and lifestyles and to develop appropriate strategies to improve their mental health.

## Supplementary Information

**Additional file 1:Table S1.** Participants’ sociodemographic characteristics. **Table S2.** Logistic regression analysis of factors associated with the experience of depressive symptoms.

## Data Availability

The datasets generated and/or analyzed during the current study are available on the Korea Disease Control Agency website [http://www.kdca.go.kr/yhs/home.jsp].

## Abbreviations

ANOVA: Analysis of variance
aOR: Adjusted odds ratio
CI: Confidence interval
KDCA: Korean Disease Control and Prevention Agency
KYRBS: Korean Youth Risk Behavior Web-based survey
OR: Odds ratio
SNS: Social networking services
ECW: Effort to control weight
