# Supplementary material for: Assessment of life factors affecting the experience of depressive symptoms in adolescents: a secondary analysis using the Korea Youth Risk Behavior Survey
Source: Child Adolesc Psychiatry Ment Health. 2021 Sep 24;15:50. doi: 10.1186/s13034-021-00407-0 (PMC8464092; doi:10.1186/s13034-021-00407-0)
Supplement: Supplementary file 1 — Additional file 1:Table S1. Participants’ sociodemographic characteristics. Table S2. Logistic regression analysis of factors associated with the experience of depressive symptoms. [file 13034_2021_407_MOESM1_ESM.docx]

Additional file 1:

Table S1. Participants’ sociodemographic characteristics

| Variables | Grade 7  (*n*: 7975) | Grade 8  (*n*: 7723) | Grade 9  (*n*: 8014) | Grade 10  (*n*: 7498) | Grade 11  (*n*: 7242) | Grade 12  (*n*: 7754) | χ^2^ or *F* | *p*-value |
| --- | --- | --- | --- | --- | --- | --- | --- | --- |
| Age, (in years) | 12.50±0.51 | 13.52± 0.52 | 14.51± 0.52 | 15.51± 0.52 | 16.50± 0.52 | 17.47± 0.52 |  |  |
| Gender  Boys  Girls | 4096 (51.4)  3879 (48.6) | 3920 (50.8)  3803 (49.2) | 4149 (51.8)  3865 (48.2) | 3879 (51.7)  3619 (48.3) | 3698 (51.1)  3544 (48.9) | 4031 (52.0)  3723 (48.0) | 3.41 | 0.637 |
| Size of the city  Big  Medium  Small | 3476 (43.6)  3880 (48.7)  619 (7.8) | 3334 (43.2)  3774 (48.9)  615 (8.0) | 3502 (43.7)  3917 (48.9)  595 (7.4) | 3408 (45.5)  3489 (46.5)  601 (8.0) | 3248 (44.8)  3427 (47.3)  567 (7.8) | 3564 (46.0)  3596 (46.4)  594 (7.7) | 24.65 | 0.006 |
| School type  Co-educational school  Boys school  Girls school | 5878 (73.7)  1079 (13.5)  1018 (12.8) | 5664 (73.3)  1074 (13.9)  985 (12.8) | 5900 (73.6)  1095 (13.7)  1019 (12.7) | 4377 (58.4)  1532 (20.4)  1589 (21.2) | 4204 (48.1)  1498 (20.7)  154 (21.3) | 4491 (57.9)  1604 (20.7)  1659 (21.4) | 1238.48 | < 0.01 |
| Perceived academic performance  Upper  Upper middle  Middle  Lower middle  Lower | 1468 (18.4)  2493 (31.3)  2509 (31.5)  1143 (14.3)  362 (4.5) | 1183 (15.3)  2068 (26.8)  2238 (29.0)  1616 (20.9)  618 (8.0) | 1323 (16.5)  2083 (26.0)  2093 (26.1)  1768 (22.1)  747 (9.3) | 737 (9.8)  1753 (23.4)  2343 (31.2)  1861 (24.8)  804 (10.7) | 702 (9.7)  1665 (23.0)  2379 (32.9)  1773 (24.5)  723 (10.0) | 829 (10.7)  1857 (23.9)  2521 (32.5)  1844 (23.8)  703 (9.1) | 1137.55 | < 0.01 |
| Multicultural family^a^  Yes  No | 170 (2.8)  5803 (97.2) | 130 (2.4)  5213 (97.6) | 103 (1.9)  5213 (98.1) | 62 (1.3)  4617 (98.7) | 58 (1.3)  4256 (98.7) | 57 (1.3)  4371 (98.7) | 60.16 | < 0.01 |
| Type of residence  Living with parents  Living with relatives  Living alone  Living in a dormitory  Living in a childcare facility | 7813 (98.0)  42 (0.5)  11 (0.1)  91 (1.1)  18 (0.2) | 7599 (98.4)  28 (0.4)  15 (0.2)  61 (0.8)  20 (0.3) | 7884 (98.4)  28 (0.3)  19 (0.2)  57 (0.7)  26 (0.3) | 6799 (90.7)  35 (0.5)  36 (0.5)  606 (8.1)  22 (0.3) | 6660 (92.0)  39 (0.5)  60 (0.8)  463 (6.4)  20 (0.3) | 7147 (92.2)  38 (0.5)  67 (0.9)  479 (6.2)  23 (0.3) | 1289.57 | < 0.01 |
| Perceived household economic status  Upper  Upper middle  Middle  Lower middle  Lower | 1303 (16.3)  2635 (33.0)  3425 (42.9)  542 (6.8)  70 (0.9) | 965 (12.5)  2350 (30.4)  3638 (47.1)  661 (8.6)  109 (1.4) | 874 (10.9)  2344 (29.2)  392 (48.8)  751 (9.4)  133 (1.7) | 652 (8.7)  2071 (27.6)  3761 (50.2)  842 (11.2)  172 (2.3) | 597 (8.2)  1894 (26.2)  3615 (49.9)  933 (12.9)  203 (2.8) | 578 (7.5)  1946 (25.1)  3952 (51.0)  1055 (13.6)  223 (2.9) | 975.18 | < 0.05 |
| Physical activity^d^, (in days) | 2.32±2.21 | 2.26±2.16 | 2.20±2.19 | 1.83±2.02 | 1.84±2.01 | 1.69±2.00 | 124.576 | < 0.01 |
| Time spent studying (WD^b^), (in min) | 376.68±242.87 | 415.80±242.87 | 425.80±237.63 | 520.05±263.81 | 516.83±251.16 | 550.70±252.37 | 605.67 | < 0.01 |
| Time spent studying (WK^c^), (in min) | 148.56±155.73 | 186.37±178.95 | 191.13±187.34 | 266.74±225.93 | 276.76±232.38 | 339.08±266.73 | 871.87 | < 0.01 |
| Duration of Internet use (WD^b^), (in min) | 111.75±112.57 | 122.82±122.45 | 117.96±120.20 | 104.29±109.48 | 103.07±110.23 | 97.73±105.12 | 55.16 | < 0.01 |
| Duration of Internet use (WK^c^), (in min) | 186.55±163.00 | 208.39±174.33 | 202.43±176.93 | 189.43±161.08 | 174.93±161.61 | 163.74±152.89 | 78.37 | < 0.01 |
| Dietary behaviors^e^, (in days^e^) | 4.28±2.74 | 4.04±2.75 | 3.95±2.77 | 4.02±2.68 | 3.83±2.68 | 4.01±2.70 | 21.96 | < 0.01 |
| Perceived health status  Very healthy  Healthy  Normal  Unhealthy  Very unhealthy | 2438 (30.6)  3736 (46.8)  1528 (19.2)  263 (3.3)  10 (0.1) | 2134 (27.6)  3544 (45.9)  1667 (21.6)  358 (4.6)  20 (0.3) | 2292 (28.6)  3527 (44.0)  1715 (21.4)  455 (5.7)  25 (0.3) | 1932 (25.8)  3401 (45.4)  1604 (21.4)  532 (7.1)  29 (0.4) | 1714 (23.7)  3151 (43.5)  1753 (24.2)  575 (7.9)  49 (0.7) | 1860 (24.0)  3200 (41.3)  1933 (24.9)  712 (9.2)  49 (0.6) | 560.04 | < 0.01 |
| Perception of body shape  Very skinny  Skinny  Average  Obese  Very obese | 332 (4.2)  1802 (22.6)  2944 (37.5)  2395 (30.0)  452 (5.7) | 336 (4.4)  1756 (22.7)  2898 (37.5)  2309 (29.9)  424 (5.5) | 359 (4.5)  1770 (22.1)  2965 (37.0)  2465 (30.8)  455 (5.7) | 341 (4.5)  1582 (21.1)  2680 (35.7)  2399 (32.0)  496 (6.6) | 293 (4.0)  1465 (20.2)  2572 (35.5)  2682 (34.6)  514 (7.1) | 285 (3.7)  1480 (19.1)  2727 (35.2)  2682 (34.6)  580 (7.5) | 145.23 | < 0.01 |
| Efforts to control weight  No  Weight loss  Weight gain  Weight maintenance | 3553 (44.6)  2581 (32.4)  561 (7.0)  1280 (16.1) | 3577 (46.3)  2610 (33.8)  536 (6.9)  1000 (12.9) | 3648 (45.5)  2752 (34.3)  655 (8.2)  959 (12.0) | 3618 (48.3)  2499 (33.3)  572 (7.6)  809 (10.8) | 3431 (47.4)  2547 (35.2)  537 (7.4)  727 (10.0) | 4016 (51.8)  2352 (30.3)  519 (6.7)  867 (11.2) | 250.42 | < 0.01 |
| Alcohol use^+^, (yes) | 1499 (18.8) | 1951 (25.3) | 2628 (32.8) | 3052 (40.7) | 3692 (51.0) | 4549 (58.7) | 3832.16 | < 0.01 |
| Smoking^+^, (yes) | 200 (2.5) | 551 (7.1) | 926 (11.6) | 1082 (14.4) | 1335 (18.4) | 1527 (19.7) | 1595.40 | < 0.01 |
| Substance use^+^, (yes) | 41 (0.5) | 67 (0.9) | 68 (0.8) | 48 (0.6) | 43 (0.6) | 57 (0.7) | 11.31 | 0.046 |
| Sexual activity^+^, (yes) | 121 (1.5) | 172 (2.2) | 276 (3.4) | 374 (5.0) | 549 (7.6) | 770 (9.9) | 884.35 | < 0.01 |
| Violence^f^, (yes) | 131 (1.6) | 166 (2.1) | 157 (2.0) | 103 (1.4) | 136 (1.9) | 135 (1.7) | 15.76 | < 0.01 |
| Perceived level of stress  Very much  A lot  A little  Not much  Not at all | 643 (8.1)  2021 (25.3)  3276 (41.1)  1625 (20.4)  410 (5.1) | 754 (9.8)  2156 (27.9)  3204 (41.5)  1307 (16.9)  302 (3.9) | 820 (10.2)  2143 (26.7)  3407 (42.5)  1353 (16.9)  291 (3.6) | 822 (11.0)  2166 (28.9)  3203 (42.7)  1044 (13.9)  263 (3.5) | 874 (12.1)  2175 (30.0)  2991 (41.3)  989 (13.7)  213 (2.9) | 976 (12.6)  2264 (29.2)  3223 (41.6)  1057 (13.6)  234 (3.0) | 388.48 | < 0.01 |
| Depressive symptoms, (yes) | 1752 (22.0) | 2075 (26.9) | 2228 (27.8) | 1993 (26.6) | 2024 (27.9) | 2294 (29.6) | 134.79 | < 0.01 |

Each number is presented as the mean (standard deviation) or sample size (ratio by group). Multicultural family^a^: those whose father or mother was not born in South Korea WD^b^: weekday, WK^c^: weekend, Physical activity: Number of days per week participants engaged in physical activity for at least 60 minutes per day of the week; Dietary behavior: number of days per week on which participants ate breakfast of the week; ^+^**:** lifetime experience; Violence^f^: Experience of receiving treatment on account of being a victim of violence

Table S2. Logistic regression analysis of factors associated with the experience of depressive symptoms

| Variables | Unadjusted Model | | | |  | Adjusted Model^*^ | | | |
| --- | --- | --- | --- | --- | --- | --- | --- | --- | --- |
|  | OR | 95% Cl for OR | | p-value |  | OR | 95% Cl for OR | | p-value |
| Gender  Boys  Girls | 1  1.879 | 1.802 | 1.959 | < 0.001 |  | 1.998 | 1.881 | 2.122 | < 0.001 |
| City type  Small  Big  Medium | 1  0.987  1.058 | 0.911  0.977 | 1.070  1.146 | 0.751  0.165 |  |  |  |  |  |
| School type  Co-educational  Boys  Girls | 1  0.635  1.197 | 0.597  1.134 | 0.675  1.263 | < 0.001  < 0.001 |  | 1  0.852  0.881 | 0.792  0.827 | 0.916  0.939 | < 0.001  < 0.001 |
| School grade  Middle 1st grade  Middle 2nd grade  Middle 3rd grade  High 1st grade  High 2nd grade  High 3rd grade | 1  1.305  1.368  1.286  1.378  1.492 | 1.213  1.273  1.195  1.280  1.389 | 1.404  1.470  1.384  1.483  1.604 | < 0.001  < 0.001  < 0.001  < 0.001  < 0.001 |  | 1  1.123  1.098  0.924  0.867  0.886 | 1.038  1.015  0.849  0.795  0.812 | 1.215  1.188  1.006  0.945  0.966 | < 0.01  < 0.05  0.067  < 0.01  < 0.01 |
| Perceived academic performance  Upper  Upper middle  Middle  Lower middle  Lower | 1  1.175  1.234  1.616  2.113 | 1.092  1.150  1.501  1.935 | 1.265  1.326  1.740  2.309 | < 0.001  < 0.001  < 0.001  < 0.001 |  | 1  1.097  1.124  1.317  1.583 | 1.013  1.039  1.211  1.429 | 1.188  1.217  1.433  1.753 | < 0.05  < 0.01  < 0.001  < 0.001 |
| Multicultural family, (yes) | 0.869 | 0.718 | 1.052 | 0.150 |  |  |  |  |  |
| Type of residence  Living with parents  Living in a relative's house  Lodging or living alone  Living in a dormitory  Living in a childcare facility | 1  1.920  1.594  1.148  1.200 | 1.457  1.201  1.034  0.823 | 2.530  2.115  1.275  1.748 | < 0.001  < 0.01  < 0.05  0.343 |  | 1  1.343  1.191  1.145  0.800 | 0.987  0.877  1.020  0.515 | 1.826  1.619  1.285  1.242 | 0.060  0.263  < 0.05  0.320 |
| Perceived household economic status  Upper  Upper middle  Middle  Lower middle  Lower | 1  1.114  1.093  1.778  2.516 | 1.032  1.017  1.628  2.173 | 1.202  1.174  1.942  2.912 | < 0.001  < 0.05  < 0.001  < 0.001 |  | 1  1.015  0.902  1.250  1.509 | 0.933  0.832  1.131  1.281 | 1.104  0.978  1.381  1.779 | 0.731  < 0.05  < 0.001  < 0.001 |
| Perceived health status  Very healthy  Healthy  Normal  Unhealthy  Very unhealthy | 1  1.429  2.326  3.897  6.393 | 1.352  2.189  3.577  4.740 | 1.511  2.473  4.247  8.623 | < 0.001  < 0.001  < 0.001  < 0.001 |  | 1  1.377  2.115  3.406  6.198 | 1.297  1.975  3.095  4.474 | 1.463  2.264  3.748  8.585 | < 0.001  < 0.001  < 0.001  < 0.001 |
| Dietary behavior**^a^**  none  1 day  2 days  3 days  4 days  5 days  6 days  7 days | 1  1.145  1.020  1.007  0.983  0.904  0.820  0.671 | 1.049  0.939  0.925  0.898  0.837  0.751  0.632 | 1.248  1.107  1.095  1.076  0.977  0.894  0.712 | < 0.01  0.645  0.880  0.711  < 0.05  < 0.001  < 0.001 |  | 1  1.086  0.992  0.970  0.975  0.948  0.895  0.793 | 0.989  0.908  0.886  0.884  0.872  0.815  0.742 | 1.193  1.085  1.063  1.075  1.031  0.983  0.847 | 0.086  0.866  0.514  0.608  0.213  < 0.05  < 0.001 |
| Perception of body shape  Average  Very skinny  Skinny  Obese  Very obese | 1  1.114  1.016  1.190  1.352 | 1.003  0.960  1.132  1.241 | 1.239  1.076  1.251  1.472 | < 0.05  0.578  < 0.001  < 0.001 |  | 1  1.166  1.133  1.022  0.934 | 1.033  1.062  0.967  0.849 | 1.316  1.209  1.080  1.029 | < 0.05  < 0.001  0.434  0.168 |
| Efforts to control weight  None  Weight loss  Weight gain  Weight maintenance | 1  1.546  1.154  1.149 | 1.476  1.062  1.074 | 1.619  1.254  1.229 | < 0.001  < 0.01  < 0.001 |  | 1  1.298  1.223  1.151 | 1.230  1.111  1.070 | 1.371  1.345  1.239 | < 0.001  < 0.001  < 0.001 |
| Physical activity**^b^**  None  1 day  2 days  3 days  4 days  5 days  6 days  7 days | 1  1.136  1.007  1.014  0.943  0.951  0.985  1.054 | 1.067  0.945  0.948  0.864  0.869  0.856  0.965 | 1.209  1.073  1.085  1.030  1.040  1.134  1.151 | < 0.001  0.836  0.682  0.193  0.271  0.837  0.244 |  | 1  1.212  1.181  1.297  1.292  1.328  1.446  1.625 | 1.133  1.102  1.203  1.173  1.201  1.241  1.469 | 1.297  1.265  1.398  1.425  1.467  1.685  1.797 | < 0.001  < 0.001  < 0.001  < 0.001  < 0.001  < 0.001  < 0.001 |
| Time spent studying (WD**^c^**)  360–479 min  < 240 min  240–359 min  480–599 min  600–719 min  > 720 min | 1  0.990  1.079  1.022  1.077  1.178 | 0.920  0.986  0.949  0.999  1.095 | 1.065  1.182  1.101  1.161  1.268 | 0.777  0.100  0.565  0.052  < 0.001 |  | 1  1.149  1.194  1.015  1.062  1.125 | 1.062  1.083  0.937  0.978  1.033 | 1.243  1.316  1.099  1.154  1.226 | < 0.01  < 0.001  0.715  0.153  < 0.01 |
| Time spent studying (WK**^d^**)  None  1–119 min  120–239 min  240–359 min  360–479 min  > 480 min | 1  0.876  0.928  0.991  0.985  1.121 | 0.812  0.864  0.919  0.901  1.039 | 0.944  0.996  1.070  1.077  1.210 | < 0.001  < 0.05  0.824  0.744  < 0.01 |  | 1  1.014  1.102  1.217  1.267  1.518 | 0.934  1.018  1.116  1.144  1.380 | 1.100  1.193  1.327  1.404  1.670 | 0.743  < 0.05  < 0.001  < 0.001  < 0.001 |
| Duration of internet use (WD**^c^**)  None  1–60 min  61–120 min  121–180 min  > 180 min | 1  0.885  0.954  1.057  1.344 | 0.834  0.897  0.987  1.259 | 0.940  1.014  1.132  1.434 | < 0.001  0.134  0.115  < 0.001 |  | 1  1.025  1.057  1.042  1.174 | 0.949  0.974  0.950  1.068 | 1.107  1.147  1.144  1.292 | 0.531  0.184  0.381  < 0.05 |
| Duration of internet use (WK**^d^**)  None  1–60 min  61–120 min  121–180 min  > 180 min | 1  0.799  0.818  0.817  1.000 | 0.739  0.761  0.762  0.947 | 0.865  0.879  0.877  1.057 | < 0.001  < 0.001  < 0.001  0.993 |  | 1  0.866  0.895  0.894  0.929 | 0.788  0.819  0.817  0.854 | 0.952  0.979  0.979  1.010 | < 0.01  < 0.05  < 0.05  < 0.01 |
| Alcohol use^+^, (yes) | 1.889 | 1.811 | 1.970 | < 0.001 |  | 1.683 | 1.600 | 1.770 | < 0.001 |
| Smoking^+^, (yes) | 1.911 | 1.803 | 2.025 | < 0.001 |  | 1.516 | 1.411 | 1.629 | < 0.001 |
| Substance use^+^, (yes) | 2.794 | 2.245 | 3.477 | < 0.001 |  | 1.738 | 1.353 | 2.232 | < 0.001 |
| Sexual activity, (yes) | 1.996 | 1.831 | 2.176 | < 0.001 |  | 1.504 | 1.360 | 1.663 | < 0.001 |
| Violence^e^, (yes) | 2.459 | 2.142 | 2.823 | < 0.001 |  | 2.149 | 1.839 | 2.510 | < 0.001 |

^*^Adjusted Model for all variables shown in Supplementary Table 2, except for the multicultural family and city type. Dietary behavior: Number of days per week on which participants ate breakfast, physical activity b: number of days per week participants engaged in physical activity for at least 60 minutes, WD^c^: weekday, WK^d^: weekend, Violence^e^: Experience of receiving treatment on account of being a victim of violence, ^+^: lifetime experience, CI: confidence interval, OR: odds ratio
